# Improving medical reasoning through retrieval and self-reflection with retrieval-augmented large language models

**DOI:** 10.1093/bioinformatics/btae238

**Published:** 2024-06-28

**Authors:** Minbyul Jeong, Jiwoong Sohn, Mujeen Sung, Jaewoo Kang

**Affiliations:** Department of Computer Science, Korea University, Seoul 02841, Republic of Korea; Department of Computer Science, Korea University, Seoul 02841, Republic of Korea; Department of Software Convergence, School of Computing, Kyung Hee University, Republic of Korea; Department of Computer Science, Korea University, Seoul 02841, Republic of Korea; AIGEN Sciences, Seoul 04778, Republic of Korea

## Abstract

**Summary:**

Recent proprietary large language models (LLMs), such as GPT-4, have achieved a milestone in tackling diverse challenges in the biomedical domain, ranging from multiple-choice questions to long-form generations. To address challenges that still cannot be handled with the encoded knowledge of LLMs, various retrieval-augmented generation (RAG) methods have been developed by searching documents from the knowledge corpus and appending them unconditionally or selectively to the input of LLMs for generation. However, when applying existing methods to different domain-specific problems, poor generalization becomes apparent, leading to fetching incorrect documents or making inaccurate judgments. In this paper, we introduce **Self-BioRAG**, a framework reliable for biomedical text that specializes in generating explanations, retrieving domain-specific documents, and self-reflecting generated responses. We utilize 84k filtered biomedical instruction sets to train Self-BioRAG that can assess its generated explanations with customized reflective tokens. Our work proves that domain-specific components, such as a retriever, domain-related document corpus, and instruction sets are necessary for adhering to domain-related instructions. Using three major medical question-answering benchmark datasets, experimental results of Self-BioRAG demonstrate significant performance gains by achieving a 7.2% absolute improvement on average over the state-of-the-art open-foundation model with a parameter size of 7B or less. Similarly, Self-BioRAG outperforms RAG by 8% Rouge-1 score in generating more proficient answers on two long-form question-answering benchmarks on average. Overall, we analyze that Self-BioRAG finds the clues in the question, retrieves relevant documents if needed, and understands how to answer with information from retrieved documents and encoded knowledge as a medical expert does. We release our data and code for training our framework components and model weights (7B and 13B) to enhance capabilities in biomedical and clinical domains.

**Availability and implementation:**

Self-BioRAG is available at https://github.com/dmis-lab/self-biorag.

## 1 Introduction

**Figure 1.**
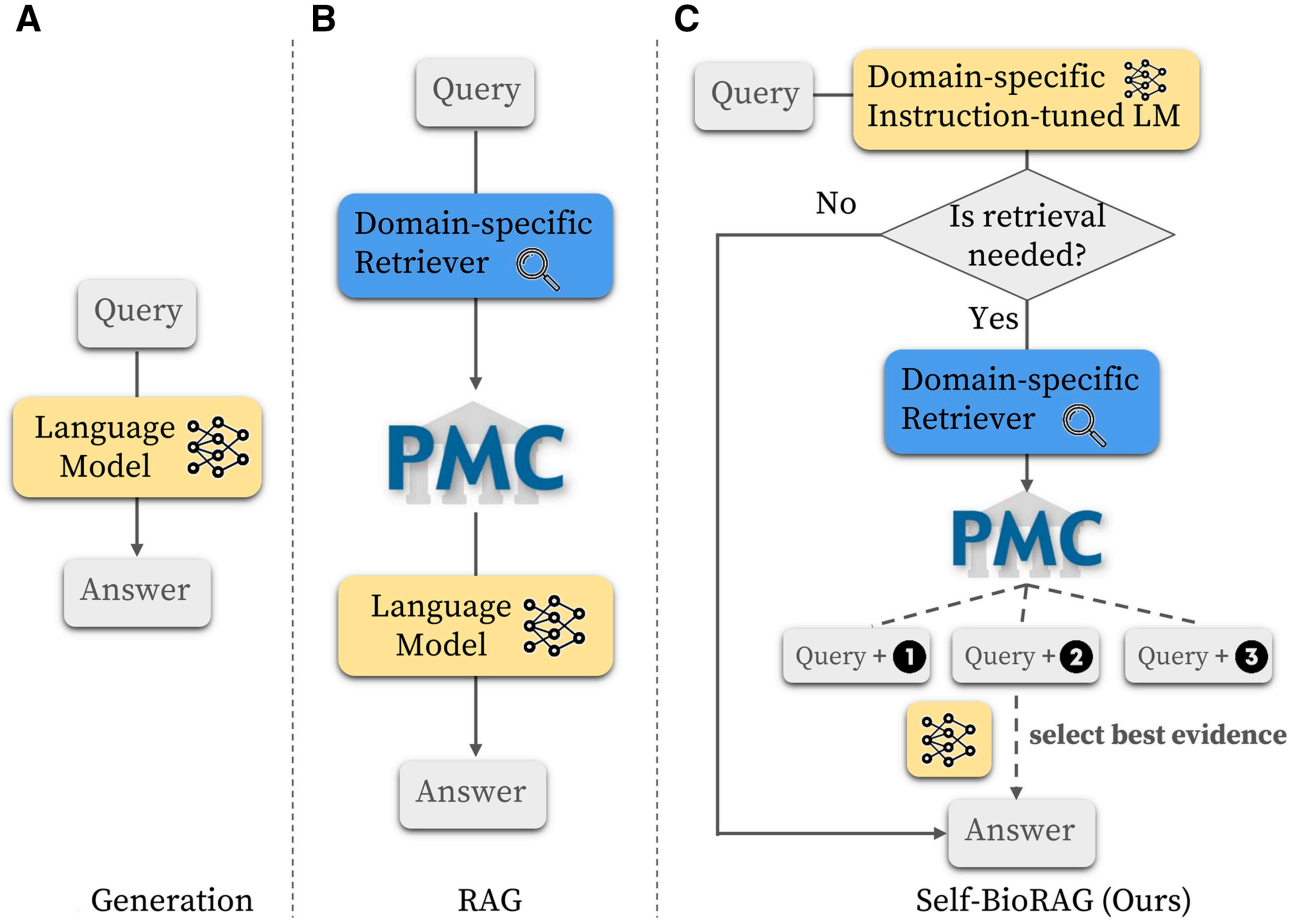
Comparison between three frameworks: generation using language model (LM), retrieval-augmented generation (RAG) using LM, and our Self-BioRAG. (A) depicts the process of sequence-to-sequence generation of LM. (B) The RAG framework first finds relevant documents from large-scale corpus such as PubMed Central and then provides the answer based on this factual content to address the shortage of scarce knowledge. (C) Initially, our domain-specific instruction-tuned model predicts whether retrieval is necessary. If a query does not require any retrieval of knowledge (factual content), it directly predicts the answer. However, if the query necessitates retrieval knowledge, Self-BioRAG utilizes the domain-specific retriever (MedCPT, in our case) to retrieve relevant documents. After retrieving the top-*k* evidence, the model selects the most pertinent evidence for the query. Ultimately, our language model is employed to select the best evidence and generate the answer based on the selected evidence and encoded knowledge.

The recent proprietary large language models (LLMs) such as ChatGPT (OpenAI 2023a), GPT-4 (OpenAI 2023b), and BARD (Google 2023) have succeeded in reaching near or comparable levels to human experts in solving many challenging problems, ranging from multi-choice question answering to long-form text generations. While these models exhibit high efficiency and demonstrate their versatility in various domains, they fall short in comprehensively covering user-dependent information such as patient reports with encoded knowledge. These limitations can result in a groundless statement and inadvertent generation of false information, commonly known as the hallucination issue (Cao *et al.* 2022, Singhal *et al.* 2022, Wei *et al.* 2022, Ji *et al.* 2023). To address this challenge, retrieval-augmented generation (RAG) enhances explainability for readers by supplying supporting facts that underpin the responses generated by LLMs (Guu *et al.* 2020, Lewis *et al.* 2020). As illustrated in Fig. 1, various RAG frameworks search documents from the knowledge corpus such as Wikipedia and appending them unconditionally or selectively to the input of LLMs for generation. In alignment with this approach, the authors of Asai *et al.* (2023) introduce Self-RAG, which uses reflective tokens that learn to reflect on its generation process given a task input possessing the following capabilities: deciding when to use on-demand retrieval, assessing whether retrieved evidence provides useful information to solve the question, criticizing whether the evidence supports the answer, and judging whether the answer is a useful response to the question. However, using Self-RAG is unsuitable for domain-specific questions like biomedical or clinical domains which shows poor generalization, leading to fetching incorrect documents or making inaccurate judgments.

In this paper, we introduce Self-BioRAG, trained with a focus on biomedical and clinical text instructions, enabling it to address corresponding instructions adeptly. It preserves generation quality and reasoning ability while incorporating on-demand retrieval and self-reflection capabilities. Note that we use the term *reasoning* to indicate that Self-BioRAG can provide explanations on answers. To build a Self-BioRAG framework, four essential components are required: (i) biomedical instruction sets, (ii) biomedical retriever, (iii) self-reflection language model, and (iv) domain-specific instruction-tuned language model. We initially construct instruction sets focused on biomedical and clinical text. In addition to the distributed MoL-instructions (Fang *et al.* 2023) and MedInstruct (Zhang *et al.* 2023), we synthetically generate an additional 18k biomedical and clinical instructions following the Self-Instruct (Wang *et al.* 2022). By combining three datasets, we could construct 120k instruction sets addressing various biomedical instructions, including information extraction, question answering, summarization, text classification, relation extraction, and multi-choice questions (Section 3.1).

Furthermore, we use the off-the-shelf MedCPT (Jin *et al.* 2023) retriever and construct biomedical corpora as follows: PubMed Abstract, PMC Full Text, Clinical Guideline, and Medical Textbook, all tailored to biomedical and clinical text (Section 3.2). The training process for the self-reflection language model and the domain-specific instruction-tuned language model is similar to Self-RAG, except that, instead of directly training instructions into the LLaMA2 (Touvron *et al.* 2023) model, we achieve better performance by training the model weights provided by Self-RAG (Section 3.3, 3.4). The goal of our work is to *construct a language model encoded with domain-specific knowledge, enabling it to autonomously assess explanations and answers it generates*.

Self-BioRAG demonstrates its effectiveness using five open-domain question-answering (QA) benchmark datasets: multi-choice QA [MedQA (Jin *et al.* 2021), MedMCQA (Pal *et al.* 2022), and MMLU (Hendrycks *et al.* 2020)] and long-form QA [LiveQA (Abacha *et al.* 2017) and MedicationQA (Abacha *et al.* 2019)]. Experimental results on the multi-choice QA datasets demonstrate that Self-BioRAG significantly outperforms open-foundation LLMs and RAG approaches with a parameter size of 7B or less. Self-BioRAG achieves a 7.2% absolute improvement compared to the state-of-the-art model. In long-form QA datasets, we notice a substantial difference in the terminologies used despite generating predictions that are similar to answers. We demonstrate that domain-specific components contribute to the performance gains, with training on domain-specific instructions showing the highest improvement. Our biomedical corpora supplement scarce knowledge, and particularly, Self-BioRAG uses appropriate documents if needed corresponding to the benchmark datasets. We further analyze that using reflective tokens to adaptively retrieve factual content is effective in solving open-domain question-answering datasets. Overall, Self-BioRAG finds the clues in the question, retrieves relevant evidence, and understands how to answer with information using encoded knowledge.

Our contributions are as follows: (i) We introduce a Self-BioRAG framework which is extensively trained on biomedical and clinical instructions. (ii) We prove that domain-specific components such as retriever, documents, and instruction sets are necessary to address its domain-related instructions. (iii) Self-BioRAG demonstrates its effectiveness in three open-domain biomedical question-answering benchmark datasets by achieving an average absolute improvement of 7.2% compared to the state-of-the-art open-foundation model with a parameter size of 7B or less. (iv) We release our biomedical instruction sets, code for training our components used in Self-BioRAG, and model weights (7B and 13B) to be more capable in biomedical and clinical domains.

## 2 Background

### 2.1 Proprietary and open language models

Instructions serve as guidelines for how language models should perform a particular task. In the commercial field, proprietary language models such as InstructGPT (Ouyang *et al.* 2022) and ChatGPT (OpenAI 2023a) have gained significant advantages in tuning through instructions. However, researchers not involved in commercial fields may face challenges in using these models due to a lack of resources. Hence, research-friendly open foundation models like the LLaMA family (Touvron *et al.* 2023), Self-instruct (Wang *et al.* 2022), and Alpaca (Taori *et al.* 2023) are released. In this regard, domain-specific language models tailored for areas such as biomedical and clinical domains, like Galactica (Taylor *et al.* 2022) and Meditron (Chen *et al.* 2023), have also been released. Our research also aims to provide labor-inexpensive methods that are easy to use in various vertical domains, including biomedical and clinical domains. Specifically, Self-BioRAG strives to develop a model capable of solving challenging tasks, ranging from multi-choice questions to long-form generations.

### 2.2 Learning with reward strategy

The proprietary language models trained with reinforcement learning from human feedback (RLHF), such as ChatGPT (OpenAI 2023a) and GPT-4 (OpenAI 2023b), excel at executing straightforward instructions (e.g. translation, code generation, and question answering) in alignment with human intent (Christiano *et al.* 2017, Schulman *et al.* 2017, Google 2023, OpenAI 2023a,b). In Self-RAG (Asai *et al.* 2023), a critic language model is employed to offer a cost-effective reward strategy compared to RLHF, utilizing reflective tokens. The critic model determines whether a given task necessitates retrieval, evaluates the appropriateness of the retrieved context, assesses if the generated rationale aligns with the retrieved context, and ultimately judges the overall utility of the output. Our Self-BioRAG follows the approach of Self-RAG to create a domain-specific critic language model that not only maintains the aforementioned capabilities but is also well-versed in biomedical text.

### 2.3 Retrieval-augmented generation

The retrieval-augmented generation (RAG) significantly enhances performance in knowledge-intensive tasks and open-domain question-answering by providing context as input to the language model (Lewis *et al.* 2020, Mao *et al.* 2021, Kang *et al.* 2023). The retriever also plays a crucial role in language models by providing evidence for pre-training and few-shot fine-tuning (Guu *et al.* 2020, Izacard *et al.* 2022a). With the recent advancements in instruction language models, the combination of retriever and language models involves either using the retriever in advance to fetch evidence or iteratively retrieving it when needed (Jiang *et al.* 2023, Shao *et al.* 2023). Our base framework, Self-RAG (Asai *et al.* 2023), deviates from these approaches by being designed to perform retrieval on-demand, resulting in better cost efficiency compared to scenarios where retrieval is always active. However, in domain-specific fields like biomedical or clinical domains, the general method of retrieving context may not be applicable. Therefore, Self-BioRAG utilizes retrieval methods and documents tailored to specific domains, retrieving meaningful context that aligns with the intended field.

## 3 Self-BioRAG

**Figure 2.**
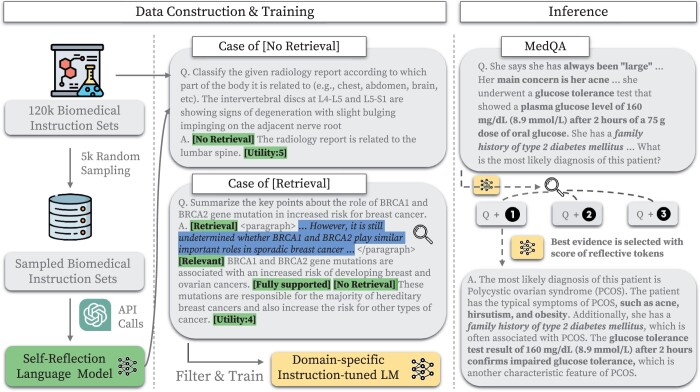
Overview of our Self-BioRAG process: data construction, training, and inference of Self-Reflection Language Model (critic LM *C*) and Domain-specific Instruction-tuned Language Model (generator LM *M*). We construct 120k biomedical instruction sets using two off-the-shelf instruction sets [Mol-Instructions (Fang *et al.* 2023) and MedInstruct (Zhang *et al.* 2023)] and one self-generated biomedical instruction set. We first sample 5k instructions to generate reflective tokens via GPT-4 API calls and then train the critic LM *C* with these instructions. Using trained critic LM *C*, we filter out mispredicted reflective tokens, such as [Continue Generation]. We preserve 84k instruction sets annotated with pre-defined reflective tokens to train the generator LM *M*. Note that critic LM *C* is only used for annotating reflective tokens used to filter instruction sets to train generator LM *M*. After training, the model *M* can predict whether or not to use the retrieval method and combine the results of evidence and encoded knowledge to answer the question. We use the MedQA (Jin *et al.* 2021) test sample to gain a proper understanding of how our Self-BioRAG works.

In this section, we outline the process of creating our Self-BioRAG framework using various biomedical components. First, we leverage three datasets consisting of biomedical instructions, which are used to train language models to align with human intentions for biomedical text (Section 3.1). To supplement scarce knowledge via relevant documents in the biomedical or clinical domains, we employ an off-the-shelf MedCPT (Jin *et al.* 2023) retriever, known for its effectiveness in retrieving relevant documents in biomedical and clinical domains (Section 3.2). Subsequently, we develop a critic language model *C* to annotate the instruction sets which will contain information for facilitating an autonomous assessment of reflective criteria (Section 3.3). Lastly, we perform training on our generator model *M* using the instruction sets created with diverse biomedical components (Section 3.4). We depict our processes of data generation, training, and inference in Fig. 2.

### 3.1 Biomedical instruction datasets

#### 3.1.1 List of instruction datasets for biomedical and clinical domains

To train the self-reflection language model (LM), also referred to as the critic LM *C*, we utilize diverse text triplets (instruction, input, output). Specifically, we collect two off-the-shelf instruction sets [Mol-Instructions (Fang *et al.* 2023) and MedInstruct (Zhang *et al.* 2023)], which include tasks like open-generation, true or false, and multi-choice questions. In addition to the distributed instruction sets, we synthetically generate an additional 18k biomedical and clinical instructions following the Self-Instruct (Wang *et al.* 2022). In total, we construct 120k biomedical instruction sets addressing diverse tasks: information extraction, question answering, and summarization. For instance, illustrated in Fig. 2, the example instruction was set to classify the given radiology report according to which part of the body is related and answer with the lumbar spine. The statistics of biomedical instruction sets are provided in Table 1. Detailed statistics of our generated instruction sets can be found in Supplementary Appendix SA.

**Table 1.** Statistics of biomedical instruction sets.^a^

Dataset name	No. of instances (original → filtered)	Tasks types
Mol-instructions (Fang *et al.* 2023)	51 493 → 38 156	Information extraction, question answering, multi-choice question
MedInstruct (Zhang *et al.* 2023)	52 002 → 36 429	Question answering, summarization, text classification, multi-choice question
Biomedical Instructions (Ours)	18 854 → 10 143	Text generation, question answering, relation extraction, text classification, summarization
Total	122 349 → 84 728	Question answering, information extraction, text classification, summarization, text generation

a We filter instructions using the critic language model *C* and use it to train the generator language model *M*.

### 3.2 Biomedical retriever

In the fields of biomedical and clinical domains, researchers and doctors addressing challenging issues typically supplement their knowledge with additional information. Similarly, for a language model to solve problems, it needs to retrieve relevant documents as needed. To achieve this, we use the off-the-shelf MedCPT (Jin *et al.* 2023) retriever (https://github.com/ncbi/MedCPT), which is contrastively trained on an unprecedented scale of 255M query-article pairs from PubMed search logs. To retrieve relevant documents, we compile data from four sources: PubMed Abstract (https://pubmed.ncbi.nlm.nih.gov/), PMC Full-text (https://www.ncbi.nlm.nih.gov/pmc/tools/textmining/), Clinical Guidelines [a publicly released subset of 35 733 guideline articles from MEDITRON (Chen *et al.*, 2023), extracted from 8 sources: CCO, CDC, CMA, ICRC, NICE, SPOR, WHO, and WikiDoc], and English Medical Textbooks [Medical textbooks widely used by medical students and takers of the United States Medical Licensing Examination (USMLE), https://github.com/jind11/MedQA]. We encode these data offline to make it computationally effective. The documents are segmented into chunks of 128 words with 32-word overlaps to form evidence following previous works (Wang *et al.* 2019, Karpukhin *et al.* 2020). We first retrieve top-*k* (*k *=* *10, in our case) evidence from each source data (total 4*k* evidence) and then use the reranking module to obtain the final top-*k* evidence relevant to the query. Table 2 presents the overall statistics of biomedical corpus and how many documents are indexed.

**Table 2.** Statistics of the indexed biomedical corpus.^a^

Data	No. of documents	No. of Chunks	Embedding size
PubMed	36 533 377	69 743 442	400 GB
PMC	1 060 173	46 294 271	160 GB
CPG	35 733	606 785	3.5 GB
Textbook	18	133 875	0.7 GB

a CPG stands for Clinical Practice Guideline.

### 3.3 Self-reflection language model (critic language model)

#### 3.3.1 Data construction of critic LM C

We collect a total of 120k biomedical instruction sets and randomly sample 5k examples (*D_s_*) to train the critic LM *C*. We use GPT-4 API Calls to generate reflective tokens *r*, guiding the critic model *C* in learning how to predict these tokens. We follow the usage of four types of reflective tokens *r* employed in Self-RAG, as described in Table 3. Detailed statistics and prompts used in generating each type of reflective token are provided in Supplementary Appendix SG. Exploring other reflective tokens suitable for specific domains is left for future work.

**Table 3.** Reflective tokens *r* used in Self-BioRAG.^a^

Type	Input	Output	Definitions
RET	x/x,y	{yes, no, continue}	Decides when to retrieve using *R*
REL	*x*, *e*	{**relevant**, irrelevant}	*e* provides useful information to solve *x*
SUP	*x*, *e*, *y*	{**fully supported**, partially supported, no support}	All of the verification-worthy statement in *y* is supported by *e*
USE	*x*, *y*	{**5**, 4, 3, 2, 1}	*y* is a useful response to *x*

a *x*, *y*, and *e* respectively indicate input, output, and evidence. Specific reflective tokens highlighted in bold are desirable during data construction as they contribute to preserving the existing instruction data possible.

#### 3.3.2 Process of training critic LM C

We observe that a critic LM released by Self-RAG (Asai *et al.* 2023)(https://github.com/AkariAsai/self-rag) is unsuitable for predicting reflective tokens in biomedical domains (see Supplementary Appendix SB for a comparison of critic LMs’ performances). Thus, we decide to develop a domain-specific critic LM *C* using our biomedical instruction sets. We split the sampled instruction sets into train and dev to train and assess the performance of the critic LM *C*. We train the model using four types of reflective tokens *r* annotated with GPT-4 API calls. We initialize the critic LM *C* with a pre-trained language model [here we use LLaMA2 (Touvron *et al.* 2023)] and train it on the sampled dataset *D_s_* to maximize the likelihood as below.

(1) $$\underset{C}{\max}\;E_{(x,y,r)∼D_{s}}\;\text{log}\;\;p_{C}(r|x,y)$$

Note that training the critic LM *C* signifies it to predict pre-defined reflective tokens given instruction, output, and optionally evidence. We use trained LM *C* to annotate whole instruction sets and filter out instances when it mispredicts the reflective tokens that are not pre-defined such as [Continue Generation]. We provide detailed hyperparameters used to train the critic LM *C* in Supplementary Appendix SC.

#### 3.3.3 Annotating biomedical instruction sets using critic LM C

After training, the model *C* predicts four types of reflective tokens: (i) identifying whether a question requires retrieval (RET); (ii) determining if retrieved evidence provides useful information to solve a question (REL); (iii) assessing whether all statements of answers can be supported by evidence (SUP); (iv) evaluating whether all statements of answers are a useful response to the question (USE). For example, in Fig. 2, the model *C* predicts the retrieval of factual content related to the role of BRCA1 and BRCA2 gene mutation ([Retrieval]). Then, the model predicts that the retrieved evidence provides a fact that BRCA1 and BRCA2 play similar roles in breast cancer and sporadic cancer ([Relevant]). By comparing a statement of the answer and retrieved evidence, the model *C* predicts that the answer could be supported by evidence ([Fully supported]). Finally, the model *C* suggests that all statements of answers are useful responses to the question ([Utility: 4]). After annotating each type of reflective token, we aggregate all results to construct a complete instance as above. We provide detailed instructions to annotate the biomedical instruction dataset using the critic LM in Supplementary Appendix SH.

### 3.4 Domain-specific instruction-tuned language model (generator language model)

#### 3.4.1 Data construction using critic LM C and training generator LM M

We use MedCPT to retrieve top-*k* evidence following an instruction that necessitates retrieval of biomedical context. After retrieving relevant documents, we use the critic LM *C* to predict each reflective token as described in Table 3. Consequently, we preserve 84k filtered instances of biomedical instruction sets annotated with pre-defined reflective tokens, instruction, and output triplets to train generator LM *M*. We want to point out that the critic LM *C* is only used to annotate reflective tokens to generate biomedical instruction sets to train generator LM *M*. We fine-tune these filtered 84k biomedical instructions on the generator model to predict answer with reflective tokens as below,

(2) $$\underset{M}{\max}\;E_{(x,y,r)∼D}\;\text{log}\;\;p_{M}(y,r|x)$$

where *D* stands for filtered instruction sets annotated with pre-defined reflective tokens *r*. This enhances generalizability in the biomedical and clinical domains preserving the abilities of text generation and self-assessment of its generated explanations with reflective tokens.

#### 3.4 2 Inference process of Self-BioRAG

In Fig. 2, we present a MedQA (Jin *et al.* 2021) example to illustrate our Self-BioRAG inference offline. For instance, the question is inquiring about the diagnosis of a female patient exhibiting symptoms of obesity, acne, and has a history of type 2 diabetes mellitus. The generator model *M* determines the need to retrieve a relevant document and selects the best evidence from the top-*k* retrieved documents based on a score *S*, calculated as the weighted sum of reflective tokens, using the same hyperparameters as Self-RAG,

$$\begin{array}{c} S(\mathrm{Critique})=\sum_{G\in G}w^{G}s^{G},\;G=\mathrm{REL}\cup \mathrm{SUP}\cup \mathrm{USE}\\ s^{G}=\frac{p(\hat{r})}{\sum_{i=1}^{N^{G}}p(r_{i})} \end{array}$$

where *s^G^* denotes the generation probability of the most desirable reflective token $\hat{r}$ (e.g. [Fully supported]) for reflective token type *G* (e.g. SUP) and *w^G^* represents the hyperparameter providing weight for *s^G^*. We can set the weight *w^G^* to adjust our behavior at inference time. For example, to find the most relevant document *e* related to question *x*, we can set a weight term *REL* score higher. Self-BioRAG is tailored to conditionally generate text without any additional training which could need balancing the trade-off between multiple preferences (Touvron *et al.* 2023, Wu *et al.* 2023b).

The prioritized evidence includes information on *the family history of type 2 diabetes mellitus* and *the patient’s diagnosis of polycystic ovarian syndrome (PCOS)*. Due to space limitations, we display partial information in the figure; please refer to the complete case in Table 8. Consequently, the generator model *M* generates the following text: (i) the patient has acne and obesity, typical symptoms of PCOS; (ii) the patient has a family history of type 2 diabetes mellitus, often associated with PCOS; (iii) the patient underwent a glucose tolerance test, a characteristic feature of PCOS. Overall, our Self-BioRAG identifies clues in the question, retrieves factual content if necessary, and responds with encoded knowledge, similar to how doctors approach such cases.

**Table 4.** Experimental results on biomedical benchmark datasets.^a^

		Open-domain biomedical benchmark			
Model	Params.	MedQA (Acc.)	MedMCQA (Acc.)	MMLU-Med (Acc.)	Average
*Proprietary LM*					
Med-PaLM (Singhal *et al.* 2022)	540B	60.3	56.5	75.6	64.1
GPT-3.5 (OpenAI 2023a)		53.6	51.0	67.3	57.2
GPT-4-base (OpenAI 2023b)		86.1	73.7	89.9	83.2
*Open LM*					
Alpaca (Taori *et al.* 2023)	7B	23.6	30.4	34.4	29.5
FLAN-T5 (Chung *et al.* 2022)	3B	36.1	20.0	44.2	33.4
PMC-LLaMA (Wu *et al.* 2023a)	7B	26.7	26.5	25.8	26.3
Galactica (Taylor *et al.* 2022)	6.7B	29.5	32.7	39.4	33.9
MedAlpaca (Han *et al.* 2023)	7B	35.6	36.6	39.5	37.2
MEDITRON (Chen *et al.* 2023)	7B	36.5	37.1	41.1	38.3
LLaMA2 (Touvron *et al.* 2023)	7B	35.2	36.3	46.3	39.3
*Open LM (LLaMA2) + Retrieval*					
RAG	7B	36.2	38.3	47.7	40.7
Self-RAG (Asai *et al.* 2023)	7B	31.2	36.5	45.7	37.8
Self-BioRAG (Ours)	7B	**43.6**	**42.1**	**53.9**	**46.5**
Self-BioRAG (Ours)	13B	**48.6**	**44.0**	**57.2**	**49.9**

a We use 3-shot examples as guidelines for language models to address benchmark instances. These examples are chosen from each training dataset using k-nearest-neighbor (Guo *et al.* 2003). Since the MMLU dataset lacks training data, we employ the same examples detailed in the appendix of MedPALM (Singhal *et al.* 2022). The score of GPT-3.5 and GPT-4-base models are from the following paper (Nori *et al.* 2023). We use biomedical corpus (e.g. PubMed, PMC, CPG, and Textbook) as evidence during inference on the RAG setting. The best score is highlighted in bold for the parameter size of 7B or less and our 13B model.

**Table 5.** Results of Long-form question-answering benchmark.^a^

Model	LiveQA (R1/R2/RL/BS)	MedicationQA (R1/R2/RL/BS)
MEDITRON (Chen *et al.* 2023)	5.5/0.0/2.5/77.2	4.1/0.2/3.3/75.9
LLaMA2 (Touvron *et al.* 2023)	8.8/1.9/6.2/**78.8**	5.7/1.2/4.4/77.6
RAG	11.5/2.3/11.1/69.5	9.8/1.3/4.8/72.9
Self-BioRAG (Ours)	**19.7**/**3.1**/**13.4**/77.2	**17.6**/**3.3**/**13.5**/**80.2**

a We report the Rouge-1 (R1), Rouge-2 (R2), Rouge-L (RL) scores to measure n-gram recall performance and report BERTScore (BS) which computes the similarity of two sentences as a sum of cosine similarities between their tokens’ embeddings.

The best scores are highlighted in the bold.

**Table 6.** Effect of each domain-adaptation component.

Experiment Detail	MedQA (Acc.)	MedMCQA (Acc.)	MMLU-Med (Acc.)	Average
Self-BioRAG	**43.6**	**42.1**	**53.9**	**46.5**
– Reflective Tokens	42.5	41.9	51.1	45.2 (-1.3)
– Biomedical Corpora	40.7	40.7	49.3	43.6 (-2.9)
– MedCPT Retriever	39.8	38.9	47.6	42.1 (-4.4)
– Biomedical Instruction Sets	34.8	36.4	46.4	39.2 (-7.3)

Self-BioRAG provide highest score represented in bold.

**Table 7.** Effect of adaptive retrieval in Self-BioRAG.^a^

Methods	MedQA (Acc.)	MedMCQA (Acc.)	MMLU-Med (Acc.)	Average
Only [No Retrieval]	39.7	41.9	52.8	44.8
Only [Retrieval]	40.1	**47.2**	51.3	46.2
Adaptive Retrieval (Ours)	**43.6**	42.1	**53.9**	**46.5**

a “Only [No Retrieval]” refers to not retrieving any evidence, while “Only [Retrieval]” refers to forcing the retrieval of top 10 evidences.

The best scores are highlighted in the bold.

**Table 8.** Case report of Self-BioRAG prediction using evidence in MedQA dataset.^a^

MedQA Dataset
Query: A 27-year-old woman presents to the office with **concerns about her long struggle with her physical appearance since adolescence**. She says **she has always been “large”** and was constantly targeted by her classmates and coworkers for being so. **Her main concern at the moment is her acne and unwanted facial hair** on her upper lip, for which she often visits a local spa. She has tried numerous diet plans, exercise regimens, and cosmetic products with little to no effect. Recently, **she underwent a glucose tolerance test that showed a plasma glucose level of 160 mg/dL (8.9 mmol/L) after 2 hours of a 75 g dose of oral glucose**. **She has a family history of type 2 diabetes mellitus** and a menstrual cycle that occurs every 45 days. Her pulse is 72/min and the blood pressure is 138/80 mm Hg. On physical examination, her height is **160 cm (5 ft 3 in) and her weight is 85 kg (187 lb)**, and **she has severe inflammatory acne over the cheeks and forehead and dark coarse hairs on the back**. What is the most likely diagnosis of this patient?
Option A: Hypothyroidism
Option B: Idiopathic hirsutism
Option C: Polycystic ovarian syndrome (PCOS)
Option D: Ovarian hyperthecosis
Evidence: *Early Clinical Expressions of Insulin Resistance: The Real Enemy to Look For. Today, a very common clinical scenario is a 17-year-old* ***female with a family history of type 2 diabetes mellitus (T2DM) and hypertension in her mother****and two first-degree relatives. Three years ago she was* ***diagnosed with polycystic ovarian syndrome (PCOS)****. She is* ***overweight with her body weight increasing 10 kg****in the last 2 years. Additionally, she has acanthosis nigricans (AN) in the axillae and knuckles. Blood tests were unremarkable with a fasting plasma glucose value of 82 mg/dl and an HbA1c of 5.0%.*
Prediction: The most likely diagnosis of this patient is Option C: Polycystic ovarian syndrome (PCOS). **The patient has the typical symptoms of**
**PCOS**, **such as acne, hirsutism**, and **obesity**. Additionally, **she has a family history of type 2 diabetes mellitus**, which is often associated with
PCOS. **The glucose tolerance test result of 160 mg/dL (8.9 mmol/L) after 2 hours confirms impaired glucose tolerance**, which is another characteristic feature of PCOS.

a Retrieved evidence is written in *italics*. **Blue-colored text** comprises segments connected to key information from retrieved evidence, while **red-colored text** consists of segments tied to the model’s parametric knowledge.

## 4 Experimental details

### 4.1 Benchmark evaluations

We evaluate the downstream task performance of Self-BioRAG fine-tuned on Self-RAG. Our experiments encompass multi-choice and long-form question-answering benchmarks designed to gauge biomedical and clinical knowledge encoded in language models. Following the approach of previous work, such as MedPaLM (Singhal *et al.* 2022), we use three datasets for few-shot evaluation on open-domain question-answering: MedQA (Jin *et al.* 2021), MedMCQA (Pal *et al.* 2022), and MMLU (Hendrycks *et al.* 2020). In MMLU, we extract six clinical topics: anatomy, clinical knowledge, college biology, college medicine, medical genetics, and professional medicine. For long-form question-answering benchmarks, we use two datasets for evaluation: LiveQA (Abacha *et al.* 2017) and MedicationQA (Abacha *et al.* 2019).

### 4.2 Baselines

In Table 4, we compare Self-BioRAG with proprietary, open foundation, and open foundation with retrieval-augmented language models. We report the Med-PaLM score as presented in Med-PaLM (Singhal *et al.* 2022) and the GPT-3.5 and GPT-4-base scores as presented in Nori *et al.* (2023) to establish the upper bound of benchmark datasets (Row 1–3). Open foundation models, pre-trained for sequence-to-sequence generation with instruction tuning, such as Alpaca (Taori *et al.* 2023) and Flan-T5 (Chung *et al.* 2022), are reported (Rows 4 and 5), as well as models fine-tuned on the specific vertical domains (e.g. biomedical and clinical), like PMC-LLaMA (Wu *et al.* 2023a), Galactica (Taylor *et al.* 2022), MedAlpaca (Han *et al.* 2023), and Meditron (Chen *et al.* 2023) (Row 6–9). LLaMA2 (Touvron *et al.* 2023) demonstrates state-of-the-art performance in open-foundation 7B models in our experiment (Row 10). Therefore, we employ LLaMA2 for the result of retrieval-augmented generation (RAG) and provide the top-10 evidence collected from the biomedical corpus using the MedCPT retriever (Row 11). Due to the length limit of RAG for input, we can only leverage the top 1 evidence in input and few-shot examples. In addition, we report Self-RAG (Asai *et al.* 2023) using Contriever (Izacard *et al.* 2022b) fine-tuned on MSMARCO (Bajaj *et al.* 2016) with the Wikipedia corpus (Row 12). We compare these baselines with our Self-BioRAG framework which is trained with biomedical components.

### 4.3 Training and inference settings

Self-BioRAG is trained with 84k biomedical instruction sets filtered using a trained critic language model (LM). We adopt the Self-RAG critic LM as our base model and fine-tune it with 5k sampled instruction sets annotated by GPT-4 API calls. As training on the Self-RAG generator LM yields better results, we fine-tune our biomedical instruction sets instead of training directly on LLaMA2 (Touvron *et al.* 2023) or Meditron (Chen *et al.* 2023). For the retriever, we use the off-the-shelf MedCPT (Jin *et al.* 2023) retriever, specialized in retrieving documents based on biomedical queries and retrieving up to ten evidence for each input.

For inference, we use vllm (Kwon *et al.* 2023) to speed up our inference time. Following Self-RAG (Asai *et al.* 2023), we assign the same weight terms for reflective tokens (e.g. REL, SUP, USE) in decoding. We adopt adaptive retrieval by default which dynamically decides when to retrieve the evidence by predicting a reflective token [Retrieval]. we retrieve the top ten evidence from the biomedical corpus processed offline. We provide details of the retrieved percentage of source data used to evaluate biomedical benchmark datasets in Section 5.2.

## 5 Results and analysis

### 5.1 Experimental results

#### 5.1.1 What contributes to the performance improvements in Self-BioRAG?

In Table 4, we compare our Self-BioRAG with open foundation language model (LM) and retrieval augmented generation (RAG). With a parameter size of 7B or less, our Self-BioRAG outperforms other open foundation LMs (Row 4–10) in all three biomedical benchmark datasets (MedQA, MedMCQA, and MMLU-Med). We also compare our model with baselines using retrieval evidence. The RAG pipeline faces two challenges: it struggles to identify crucial evidence and encounters limitations in incorporating numerous pieces of evidence due to constraints on input length. However, our Self-BioRAG outperforms the RAG baseline and can prioritize important evidence via the values of reflective tokens, which is useful for analyzing all the retrieved evidence (Rows 11 and 13). Although Self-RAG is fine-tuned on LLaMA2, we observe that Self-RAG cannot generalize to biomedical benchmark datasets, resulting in a performance drop (Rows 10 and 12). By providing a biomedical critic LM and corpus to train a biomedical generator LM, our Self-BioRAG achieves state-of-the-art performance on 7B parameters in MedQA, MedMCQA, and MMLU-Med datasets. We also provide the 13B performance of our Self-BioRAG model to demonstrate the effectiveness of our framework works in other model parameters (Row 14). We provide the detailed performance of specific MMLU datasets in Supplementary Appendix SF.

In Table 5, we compare our Self-BioRAG with two open foundation LM by measuring n-gram recall performance [Rouge Score (Lin 2004)] and similarity of token embeddings between prediction and answer [BERTScore (Zhang *et al.* 2019)]. We observe that although all foundation models do not generate predictions with the exact same words as the answers (lower Rouge Score), they manage to explain well with words that are as similar as possible (high BERTScore). However, these scores cannot measure whether a model has generated answers with accurate rationale, how much hallucination occurs, how much it includes crucial claims, or whether it has generated answers fluently. We leave an investigation about detailed capacities related to long-text generation for future works. We aim to analyze the step-by-step process through which our Self-BioRAG achieves its state-of-the-art performance in the following subsection.

### 5.2 Analysis

#### 5.2.1 Which domain-adaptation components show the improvements compared to Self-RAG?

In Table 6, each experiment involves sequentially reducing components in Self-BioRAG. The goal is to identify the factors that significantly contributed to the performance improvement, ultimately leading to the final performance of Self-BioRAG. First, the controllable generation using reflective tokens affects the rationale which leads to predicting an answer (Row 2). Then, we observe that using four biomedical corpora (PubMed, PMC, CPG, and Medical Textbook) to retrieve appropriate evidence shows performance improvement compared to Wikipedia evidence (Row 3). We also use domain-specific MedCPT retriever instead of the Contriever (Izacard *et al.* 2022b) fine-tuned on MSMARCO (Bajaj *et al.* 2016) (Row 4). Ultimately, the most effective approach was the collection and processing of biomedical instruction sets to create both a critic language model and a generation language model (Row 5). We recommend readers collect their domain-specific instructions to address corresponding instructions.

#### 5.2.2 In biomedical corpora, what evidence is used to solve open-domain question-answering benchmarks?

**Figure 3.**
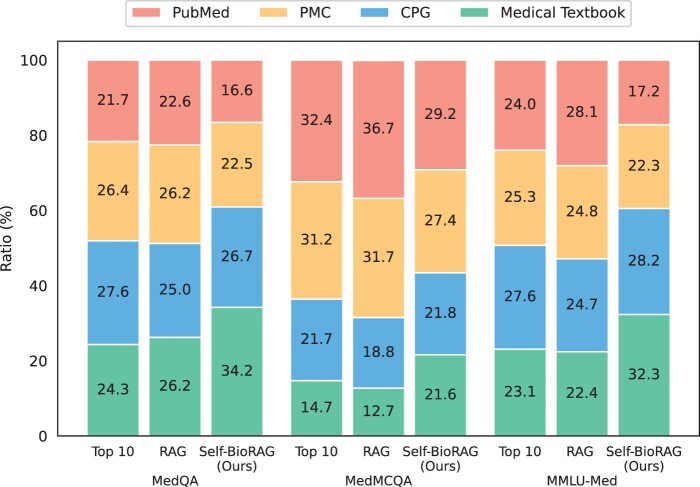
Ratio of retrieved evidences from each of the four biomedical corpora (PubMed, PMC, CPG, Medical Textbook). The RAG statistics refer to the top-1 evidence usage ratio, while Self-BioRAG selects the most useful evidence from the top-10 retrieved evidence.

In Fig. 3, we compare the ratio of retrieved evidence using the MedCPT (Jin *et al.* 2023) retriever on four biomedical corpora (PubMed, PMC, CPG, and Medical Textbook). Even though the index sizes of Medical Textbook and CPG are much smaller than PubMed or PMC, retrieved evidences show even distribution. Specifically, our Self-BioRAG only retrieves small portions to solve three datasets [MedQA (12%), MedMCQA (8%), and MMLU-Med (11%)] meaning that these open-domain benchmarks do not require that much evidence than expected. We depict these portions up to 100% in Fig. 3. We observe a trend in which Self-BioRAG retrieves a higher proportion of information from the Medical Textbook, similar to the approach used in solving USMLE-style questions. This is also aligned with previous facts that retrieving documents from Medical Textbook can achieve higher performance in clinical questions (Li *et al.* 2023, Wang *et al.* 2023).

#### 5.2.3 Does evidence truly help to supplement limited knowledge in Self-BioRAG?

In Table 7, we conduct three different experimental settings using open-domain question-answering benchmark datasets: (i) *No Retrieve*: Answer without utilizing any provided evidence. (ii) *Only Retrieve*: Retrieve top-10 evidence for each question and predict an answer. (iii) *Adaptive Retrieve*: Use a criterion to decide whether to retrieve or not based on the question. The criterion is set as follows,

(3) $$\frac{p(\mathrm{retrieve}=\mathrm{Yes})}{p(\mathrm{retrieve}=\mathrm{Yes})+p(\mathrm{retrieve}=No)}>\delta .$$

where we set *δ* hyperparameter as 0.2 for the *Adaptive Retrieve* experiment setting. Our findings indicate that retrieving relevant documents indeed aids in solving benchmark datasets. In addition, we observed that adaptively retrieving shows comparable performance on average with the *Only Retrieve* setting. This is attributed to the small portion of retrieved evidence used to answer the questions. While the *Only Retrieve* setting exhibits a substantial improvement in MedMCQA, it shows a performance drop in MMLU-Medical datasets compared to the *No Retrieve* setting, indicating its instability. As a result, we recommend readers to use the adaptive retrieval setting.

#### 5.2.4 Distinguishing when to retrieve documents in Self-BioRAG

**Figure 4.**
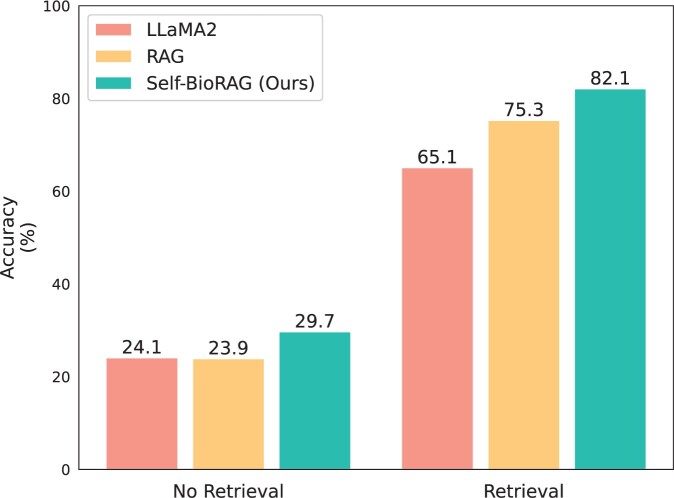
Performance of LLaMA2, RAG, and Self-BioRAG on examples split into [No Retrieval] and [Retrieval] based on Self-BioRAG using the MedQA test dataset.

In Fig. 4, we evaluate the performance of LLaMA2, RAG (LLaMA2 with MedCPT and biomedical corpora), and Self-BioRAG on examples predicted as [No Retrieval] and [Retrieval] by Self-BioRAG. To show an overall trend, we use the MedQA dataset here and the rest of the two datasets are in Supplementary Appendix SE. Notably, Self-BioRAG retrieves small portions to solve three biomedical benchmarks. Still, the results demonstrate that Self-BioRAG consistently outperforms other baselines, whether or not retrieved evidence is used. In situations where retrieval is not necessary (left column), Self-BioRAG > RAG ≈ LLaMA2. The overall trend in the retrieved situation (right column) indicates Self-BioRAG > RAG ≥ LLaMA2. Intuitively, we identify that Self-BioRAG distinguishes well on situations to use retrieved evidence or not depending on questions.

#### 5.2.5 Case report of using retrieved evidence

In Table 8, we present an example from the MedQA dataset to illustrate how Self-BioRAG works. For instance, a patient exhibits symptoms of physical appearance changes, acne, and a family history of type 2 diabetes mellitus (T2DM). Self-BioRAG determines the need to retrieve relevant documents containing information on a female diagnosed with polycystic ovarian syndrome (PCOS) and similar symptoms (e.g. T2DM and obesity). Self-BioRAG determines the patient’s diagnosis as PCOS by integrating all three: patient’s symptoms, retrieved evidence, and parametric knowledge. Throughout the query, evidence, and prediction, we color-code using blue and red to distinguish two categories of related snippets: (i) key information extracted from retrieved evidence and (ii) the model’s essential parametric knowledge, both of which are leveraged by Self-BioRAG to address the given problem. Overall, Self-BioRAG finds the clues in the question, retrieves relevant documents if needed, and understands how to answer with information of retrieved evidence and encoded knowledge same as a medical expert would do.

## 6 Conclusion

In this manuscript, we introduce the Self-BioRAG framework, enabling a Self-RAG (Asai *et al.* 2023) to generalize to biomedical and clinical domains of instructions. This framework enhances the generation capacity, facilitates the retrieval of factual content on demand, and enables self-assessment of generated rationales. Our experimental results cover five open-domain question-answering (QA) datasets widely used in biomedical and clinical domains. In multi-choice QA datasets, Self-BioRAG achieves a 7.2% absolute improvement compared to the state-of-the-art model among the open foundation 7B models. In Long-form QA datasets, Self-BioRAG exhibits notable variations in term usage, despite producing predictions that closely resemble answers. We demonstrate the necessity of domain-specific components, such as retriever, domain-related document corpus, self-reflection model, and generator model, to address domain-related instructions. We provide diverse analyses: (i) self-BioRAG retrieves a larger portion of evidence from Medical Textbook than other corpora to solve USMLE-style questions; (ii) self-BioRAG can distinguish when to retrieve evidence depending on instruction and question; (iii) provided evidence from biomedical corpora genuinely helps supplement scarce knowledge. In future works, we aim to explore generating long-form text in a fine-grained evaluation which could interpret how open foundation models (with or without domain adaptation) generate.

## Supplementary Material

btae238_Supplementary_Data
