# Supplementary material for: Improving medical reasoning through retrieval and self-reflection with retrieval-augmented large language models
Source: Bioinformatics. 2024 Jun 28;40(Suppl 1):i119–29. doi: 10.1093/bioinformatics/btae238 (PMC11211826; doi:10.1093/bioinformatics/btae238)
Supplement: btae238_Supplementary_Data [file btae238_supplementary_data.pdf]

Supplementary data for

Improving Medical Reasoning through Retrieval and Self-Reflection with Retrieval-Augmented Large Language Models

Minbyul Jeong[1], Jiwoong Sohn[1], Mujeen Sung[2,*], and Jaewoo Kang [1,3,*]

[1]Department of Computer Science, Korea University, Seoul 02841, Republic of Korea
[2]School of Computing, Kyung Hee University, Gyeonggi-do 17104, Republic of Korea
[3]AIGEN Sciences, Seoul 04778, Republic of Korea

*To whom correspondence should be addressed.

Availability and implementation: Self-BioRAG is available at https://github.com/dmis-lab/self-biorag
Contact: mujeensung@khu.ac.kr, kangj@korea.ac.kr

A Statistics of our generated Biomedical Instruction Set

In Figure 1, we depict a ROUGE-L score distribution and show that more various instructions were generated relative to the biomedical seed tasks. We compute the ROUGE-L overlap score with the seed tasks' instructions to prevent generating similar instructions as much as possible. Additionally, we observe that the generated instruction, input, and output triplets show diverse length distribution depending on the condition of instruction. In Table 1, we provide other statistics such as the average length of instructions, inputs, and outputs.

To guarantee the diverse range of instruction types, we analyze the biomedical instructions using Berkeley Neural Parser[1] (Kitaev and Klein, 2018; Kitaev *et al.*, 2019). We parse the generated instructions and extract the top 20 root verbs in instructions and their direct noun objects. Due to the requirement of the instruction-generation process, our instruction is entirely related to biomedical terms such as report, hypothesis, and diagnosis. We depict this information in Figure 2. Additionally, we provide a prompt to contain a requirement of generating biomedical/medical/clinical topics of instruction. We random sample 100 instances to manually measure the category of instruction topics. Our instruction dataset covers various biomedical topics such as text generation (42%), classification (15%), symptom identification (7%), risk value prediction (6%), clinical note editing (5%), etc.

B Critic LM Performance

We split 5k instruction sets into train and dev for training and evaluating critic language model performance. In Table 2, we use GPT-4 (OpenAI, 023b) as ground-truth predictions and identify how the critic language model of Self-RAG (Asai *et al.*, 2023) and Self-BioRAG performs. Due to the uncommon domain of questions, Self-RAG exhibits lower performance compared to our Self-BioRAG.

[1] https://parser.kitaev.io/

**Statistics**	
# of total instances	18,854
avg. instruction length (words)	24.2
avg. input length (words)	35.4
avg. output length (words)	47.8

Table S1. Statistics of our generated Biomedical Instruction Set.

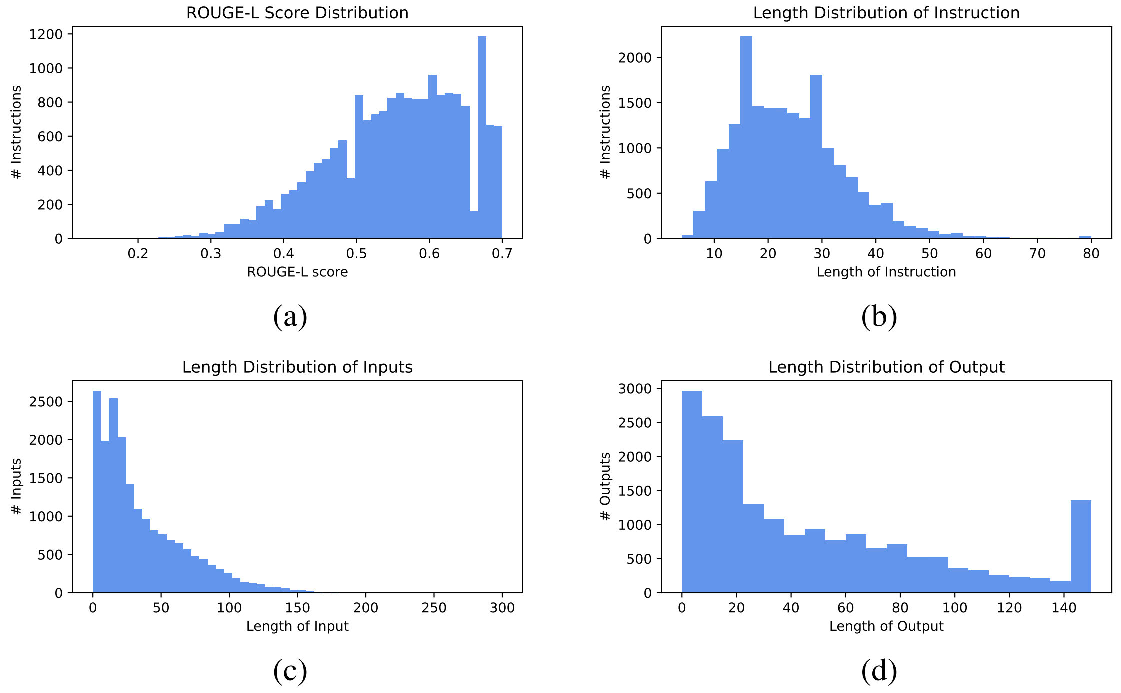

Fig. S1: (a) Distribution of ROUGE-L scores between generated instructions and their top-1 similar biomedical seed task instruction. (b) Length distribution of generated instructions. (c) Length distribution of generated inputs. (d) Length distribution of generated outputs. We set the max length of the generated output length as 150.

The critic model of Self-BioRAG trained on Self-RAG predicts reflective tokens of utility and relevance well. We find that predicting reflective tokens of retrieval and supportiveness shows far lower than we expected. However, we want to note that distilling knowledge of proprietary language models such as GPT-4 to LLaMA2 (Touvron *et al.*, 2023) can perform well in predicting reflective tokens.

**Model**	**Params.**	**Utility (Acc.)**	**Retrieval (Acc.)**	**Relevant (Acc.)**	**Supportive (Acc.)**
Self-RAG (Asai *et al.*, 2023)	7B	78.2	65.8	88.5	69.9
Self-BioRAG (Ours)	7B	89.3	73.1	91.2	75.9
Self-BioRAG (Ours)	13B	91.2	75.3	91.8	77.2

Table S2. Critic LM performance on dev set using GPT-4 (OpenAI, 023b) predictions as ground-truth predictions.

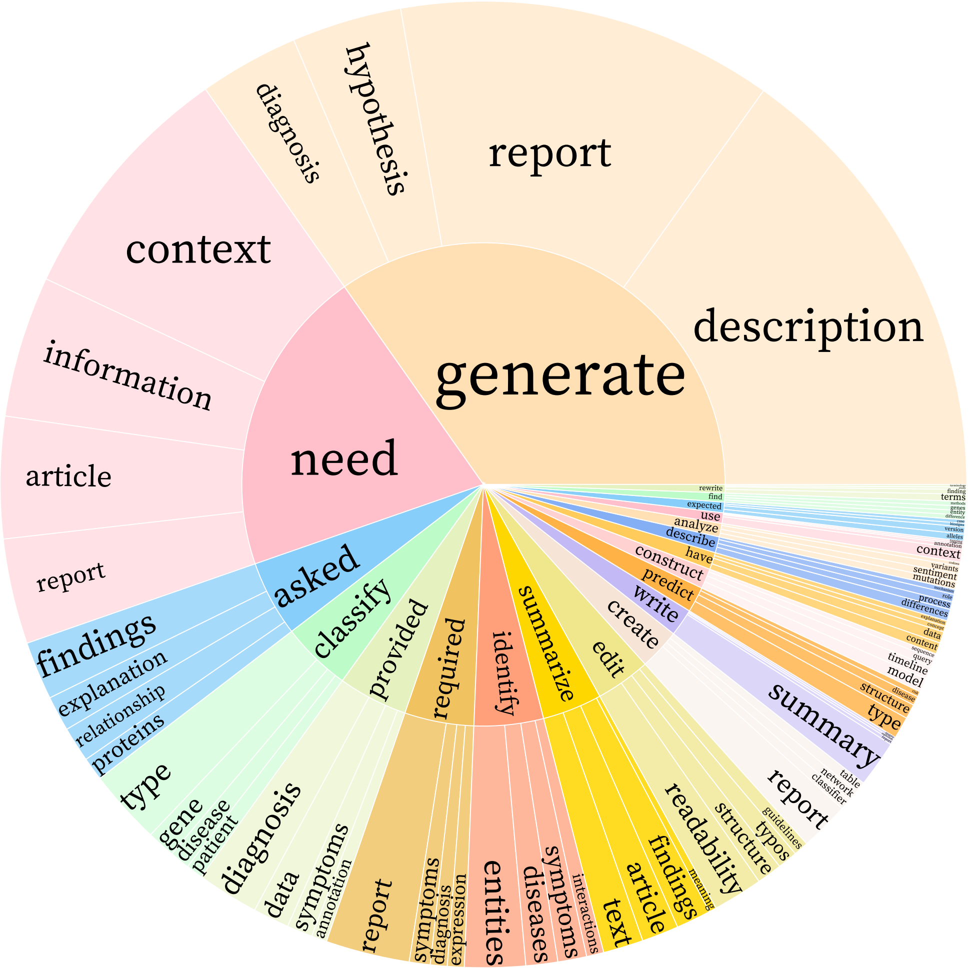

Fig. S2: Nested piechart of our biomedical instructions root verbs (inner circle) and their four noun objects (outer circle). It signifies the diversity of generated instructions and relates to biomedical terms such as hypothesis, proteins, diagnosis, and symptoms.

C Hyperparameters of Training Details

We use 8 Nvidia A100 with 80GB memory to train our Self-BioRAG models. We train our critic model for 3 epochs with a learning rate of 2e-5 with a 1% warmup rate. We also train our generator model for 5 epochs with a learning rate of 2e-5 with a 3% warmup rate, and linear decay afterward. We set our model maximum input length as 2048 for the 7B and 13B models to address at least 3 shots in training and inference. We use Deepspeed stage 3 (Rajbhandari *et al.*, 2020) to implement multi-GPU settings and FlassAttention (Dao *et al.*, 2022) for efficient training. Our code is based on Self-RAG (Asai *et al.*, 2023), which is written in PyTorch (Paszke *et al.*, 2019) and HuggingFace (Wolf *et al.*, 2019). We only try once to train our models, thus it could be useful to search for better hyperparameters of our framework.

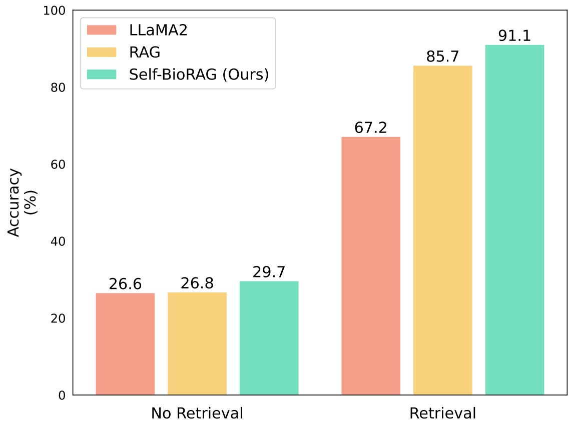

(a) MedMC QA performance

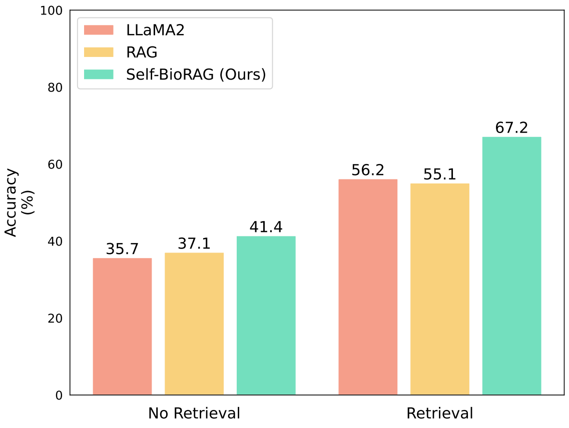

(b) MMLU-Med performance

Fig. S3: Performance of LLaMA2, RAG, and Self-BioRAG on examples predicted as [No Retrieval] and [Retrieval] by Self-BioRAG. Self-BioRAG consistently outperforms other baselines whether or not retrieved evidence is used.

D How to Set Weight of Reflective Tokens

We compute scores of each critique type as normalized probabilities of desired tokens same as Self-RAG (Asai *et al.*, 2023). For the REL token type, we compute as below.

$$S(REL) = \frac{p(relevant = yes)}{p(relevant = Yes) + p(relevant = No)} \tag{1}$$

We compute SUP using weighted scores to desirable tokens of $Fully\ supported$ as 1 and $Partially\ supported$ as 0.5. We compute our SUP token type as follows:

$$S(SUP) = \frac{p(supportive = Fully) + 0.5 * p(supportive = Partially)}{p(supportive = Fully) + p(supportive = Partially) + p(supportive = No)} \tag{2}$$

We set a weighted scores w of {-1, -0.5, 0, 0.5, 1} to the tokens of USE ={1, 2, 3, 4, 5}. We compute USE token type as follows:

$$S(USE) = \sum_{i=1}^{5} w_i \frac{p(utility = i)}{\sum_{t=1}^{5} p(utility = t)} \tag{3}$$

E Distinguishing When to Retrieve in MedMCQA and MMLU-Med Datasets

In Figure 3, we evaluate the performance of LLaMA2, RAG (LLaMA2 with MedCPT and biomedical corpora), and Self-BioRAG on examples predicted as [No Retrieval] and [Retrieval] by Self-BioRAG. We could also observe that a similar trend is found in MedMCQA (Pal *et al.*, 2022) and MMLU-Med (Hendrycks *et al.*, 2020) datasets. Experimental results demonstrate that Self-BioRAG consistently outperforms other baselines, whether or not retrieved evidence is used. Interestingly, we observe that the performance of the MedMCQA dataset shows 91.1% accuracy on retrieved questions. This also aligns with the result that the hard constraint of using retrieved evidence in all questions shows high performance compared to not retrieving any evidence or adaptively retrieving as shown in Table 6. We posit that the MedMCQA dataset contains questions requiring external knowledge to retrieve.

F Result of Closed-domain Question-answering Dataset

We evaluate Self-BioRAG on the PubMedQA (Jin *et al.*, 2019) dataset, which provides a reference context to solve questions. Our Self-BioRAG achieves 54.6% accuracy, which is slightly lower than open foundation LMs such as LLaMA2 (56.0%) or MEDITRON (58.1%). Additionally, RAG (46.2%) and Self-RAG (52.1%) exhibit lower performance in this trend. We attribute this performance drop to the use of retrieved evidence during generation. In other words, PubMedQA already provides the reference context to solve questions, and Self-BioRAG further introduces the top-10 evidence, potentially introducing errors due to excess noisy information.

G Detailed performance of MMLU-Med Datasets

To measure medical knowledge, we evaluate the Massive Multitask Language Understanding (MMLU) (Hendrycks *et al.*, 2020) dataset and extract six medical and clinical domains to construct MMLU-Med benchmark as follows: Anatomy, Clinical Knowledge, College Biology, College Medicine, Medical Genetics, and Professional Medicine. In Table 3, we provide detailed performances of the MMLU-Med benchmark. Our model Self-BioRAG outperforms other baselines used in the open foundation language model and open foundation model with language model on the MMLU-Med benchmark.

H Prompt of Data Generation for Self-Reflection Language Model

We provide prompts to generate data for the self-reflection language model (critic language model). In Table 4, utility reflective tokens are distinguished in 5 scores, ranging from 1 to 5. We use the utility to check that the output is a useful response to the instruction. In Table 5, we decide if the output can be verified solely with suggested evidence on retrieval reflective tokens. In Table 6, we use relevant reflective tokens to determine if the evidence is relevant to the instruction and the preceding context. Additionally, we use supportive reflective tokens to check an output sentence is supported by the evidence, described in Table 7.

Model	Params.	MMLU Benchmark					
		MMLU-AN	MMLU-CK	MMLU-CB	MMLU-CM	MMLU-MG	MMLU-PM
Open LM							
Alpaca	7B	44.4	35.5	23.6	31.2	37.0	34.9
FLAN-T5	3B	42.2	53.6	41.7	41.6	43.0	43.0
PMC-LLaMA	7B	35.7	21.7	26.5	23.2	28.0	19.7
Galactica	6.7B	40.7	41.5	48.6	34.1	48.0	23.5
MedAlpaca	7B	40.7	40.1	49.2	35.2	46.0	25.8
MEDITRON	7B	45.1	39.5	43.2	41.1	38.0	39.8
LLaMA2	7B	45.9	52.5	38.9	45.6	55.0	40.0
Open LM + Retrieval							
Self-RAG	7B	42.2	49.1	51.0	39.9	52.0	40.0
Self-RAG†	7B	45.2	55.1	52.8	39.9	55.0	39.7
Self-BioRAG (Ours)	7B	51.1	60.8	60.4	50.9	52.0	48.2
Self-BioRAG (Ours)	13B	51.9	60.8	66.7	51.4	60.0	52.2

Table S3. Experimental results of biomedical benchmark datasets. We use 3-shot examples to guide how to solve a benchmark example to language models. We use the same examples described in the appendix of MedPALM (Singhal et al., 2022) for the MMLU dataset. We refer to each dataset with the abbreviation of full name: Anatomy (AN), Clinical Knowledge (CK), College Biology (CB), College Medicine (CM), Medical Genetics (MG), and Professional Medicine (PM). † use biomedical corpora (e.g., PubMed, PMC, Clinical Guideline, and Medical Textbook) as evidence during inference.

I Examples of Biomedical Instructions to Train Domain-Specific Instruction-Tuned Language Model

We collect 84k biomedical instruction sets to train a domain-specific instruction-tuned language model. For example, in Table 8, we provide several cases used in training the language model: (1) Our critic language model decides to not retrieve any evidence about generating an explanation of the human cardiovascular system. (2) Retrieved evidence contains information on symptoms of Type 1 diabetes mellitus: urination, loss of weight, and stomach pain. Therefore, our critic model predicts that the output sentence (e.g., "answer is Type 1 diabetes mellitus") fully supports the evidence. (3) The critic model decides that more evidence is needed to answer the following question. Even if relevant evidence is retrieved (e.g., information about cystic fibrosis), the evidence does not support solving the original question. Overall, our critic language model predicts whether to retrieve or not based on the example instruction

References

Asai, A. *et al.* (2023). Self-rag: Learning to retrieve, generate, and critique through self-reflection. *arXiv preprint arXiv:2310.11511*.

Dao, T. *et al.* (2022). Flashattention: Fast and memory-efficient exact attention with io-awareness. *Advances in Neural Information Processing Systems*.

Hendrycks, D. *et al.* (2020). Measuring massive multitask language understanding. *arXiv preprint arXiv:2009.03300*.

Jin, Q. *et al.* (2019). Pubmedqa: A dataset for biomedical research question answering. In *Proceedings of the 2019 Conference on Empirical Methods in Natural Language Processing and*

Prompt of Reflective token type Utility (USE)
Given an instruction and an output, rate whether the response appears to be a helpful and informative answer to the query, from 1 (lowest) - 5 (highest). We call this score perceived utility.<br><br>The detailed criterion is as follows:<br>5: The response provides a complete, highly detailed, and informative response to the query, fully satisfying the information needs.<br>4: The response mostly fulfills the need in the query, while there can be some minor improvements such as discussing more detailed information, having better structure of the response, or improving coherence.<br>3: The response is acceptable, but some major additions or improvements are needed to satisfy users' needs.<br>2: The response still addresses the main request, but it is not complete or not relevant to the query.<br>1: The response is barely on-topic or completely irrelevant.<br><br>### **Instruction**: Generate a description of the structure and mechanism of action of the enzyme nicotinamide adenine dinucleotide phosphate (NADPH) oxidase.<br>**Output**: NADPH oxidase is a membrane-bound enzyme that plays an important role in the production of reactive oxygen species. It is composed of six distinct subunits and is found in both the cytosol and the membrane. The subunits are composed of two copies each of the cytosolic subunits: p47 phox, p67 phox, p40 phox, and two copies of the membrane-associated subunits: gp91phox and p22phox. NADPH oxidase functions by donating electrons to the active site of the enzyme, thus allowing it to reduce oxygen mole -cules to form superoxide anion. The end product of this reaction is the reactive oxygen species, which is used to fight infection or for other body functions. NADPH oxidase is regulated by various signals such as calcium, nicotinamide adenine dinucleotide phos -phate (NADP), and calcium binding proteins. In addition, it is inhibited by certain compounds, such as apocynin, that prevent its activation. NADPH oxidase plays an important role in the body's defenses against infection, and it is thus essential for many biolo -gical processes.<br>**Perceived utility**: 5<br>**Explanation**: This response provides a descriptive explanation of the structure and function of NADPH oxidase, giving full and comprehensive information. Thus, this is deemed a high utility response satisfying the user's information needs.<br><br>### **Instruction**: Was the enhancement of Lck activity immediate?<br>**Output**: No, the enhancement of Lck activity did not have an immediate relevance to TCR-mediated signal transduction, as judged from the lack of detectable de novo phosphorylation of zeta-chain and the absence of functional responses of T cells.<br>**Perceived utility**: 3<br>**Explanation**: The response robustly addresses the aspect of Lck activity enhancement not being immediate. However, the details provided thereafter are quite dense and technical, and without proper context, they may not be easy to interpret for all readers. Simplifying this information or providing some additional context could improve the perceived utility.<br><br>### **Instruction**: Alcohol intake during pregnancy causes, all, except?<br>**Output**: Brachycephaly<br>**Perceived utility**: 5<br>**Explanation**: This is a concise and correct answer to the posed question, focusing on the exclusion of one potential effect of alcohol intake during pregnancy. The response specifically points out that brachycephaly is not caused by alcohol intake during pregnancy, thus correctly answering the query. There's not much room for more elaborate discussion in this case, making this answer complete and highly useful for the user's information needs in this context.<br><br>### **Instruction**: {Example Instruction}<br>**Output**: {Example Output}<br>**Perceived utility**:

Table S4. Prompt of Utility Reflective token. We use 3-shots to generate reflective tokens via GPT-4 API calls.

the 9th International Joint Conference on Natural Language Processing (EMNLP-IJCNLP).

Kitaev, N. and Klein, D. (2018). Constituency parsing with a self-attentive encoder. In *Proceedings of the 56th Annual Meeting of the Association for Computational Linguistics (Volume 1: Long Papers).*

Kitaev, N. *et al.* (2019). Multilingual constituency parsing with self-attention and pre-training. In *Proceedings of the 57th Annual Meeting of the Association for Computational Linguistics*. Association for Computational Linguistics.

OpenAI (2023b). Openai gpt-4 technical report.

Prompt of Reflective token type Retrieval (RET)

You will be provided with an instruction, evidence, output sentence, and preceding sentences (optional). If the preceding sentence is given, the output should be the sentence that follows those preceding sentences. Your task is to determine whether the informat -ion in the output sentence can be fully verified by the evidence or if it requires further external verification.
If the output sentence can be verified solely with the evidence or doesn't require any verification, respond with **[No Retrieval]**.
If additional information is needed to verify the output sentence, respond with **[Retrieval]**. Please provide explanations for your judgments.

Instruction: What is the mechanism of 72T4Cl activation under physiological conditions?
Evidence: Timing mechanism and effective activation energy concerned with aging and lifespan in the long-lived and thermosens -ory mutants of Caenorhabditis elegant.. The lifespans of many poikilothermic animals, including the nematode Caenorhabditis elegans, depend significantly on environmental temperature. Using long-living, thermosensory mutants of C.elegans, we tested whether the temperature dependency of the mean lifespan is compatible with the Arrhenius equation, which typically represents one of the chemical reaction rate theories. The temperature dependency of C. elegans was the Arrhenius type or normal, but daf-2(e1370) mutants were quite different from the others. However, taking into account the effect of the thermal denaturation of DA -F-2 with the temperature, we showed that our analyzed results are compatible with previous ones. We investigated the timing me -chanism of one parameter (the onset of biodemographic aging (t(0))) in the lifespan equation by applying the RNAi feeding met -hod to daf-2 mutants in order to suppress daf-16 activity at different times during the life cycle. In summary, we further deepened the biological role of two elements, t(0) and z (the inverse of the aging rate), in the lifespan equation and mean lifespan formulated by our diffusion model z(2) = 4Dt(0), where z is composed of t(0) and D (the diffusion constant).
Output: The mechanism of 72T4Cl activation under physiological conditions is not known.
Rating: [No Retrieval]
Explanation: The output sentence does not provide any factual information that needs to be verified, it is stating a fact that the mec -hanism is not known. The evidence does not mention 72T4Cl at all, supporting the claim that the mechanism of its activation is not known.

Instruction: Pigment providing colour to stool is
Evidence: The importance of stool tests in diagnosis and follow-up of gastrointestinal disorders in children. Stool samples should be evaluated macroscopically in terms of color, consistency, quantity, form, odor, and presence of mucus. The presence of a small amount of mucus in stool is normal. However, the presence of copious mucus or bloody mucus is abnormal. The normal color is tawny due to the presence of bilirubin and bile. In infants, the stool may be green, its consistency may be watery or pasty. Stool color varies greatly depending on diet. Clay-colored or putty colored stool is observed in biliary obstructions. If more than 100 mL blood is lost from the upper gastrointestinal system, black, tarry stool is observed. Besides bleeding, black-colored stool may also be observed due to iron or bismuth treatment. Red-colored stool is observed in lower gastrointestinal tract bleeding.
Output: Stercobilinogen. The characteristic brown colour of feces is due to the presence of pigment stercobilin (derived from stercobilinogen). So, actually it is stercobilin (not stercobilinogen) which is responsible for the brown color of feces. Stercobilin is derived from stercobilinogen and therefore stercobilinogen is the best answer amongst the options provided.
Rating: [Retrieval]
Explanation: The provided evidence does not mention stercobilin or stercobilinogen providing the color to stool, although it does mention bilirubin and bile contribute to the color. Therefore, additional evidence would be necessary to verify this output sentence.

Instruction: {Example Instruction}
Preceding sentences: {Example Preceding sentences}
Evidence: {Example Evidence}
Output: {Example Output}
Rating:

Table S5. Prompt of Retrieval Reflective token. We use 2-shots to generate reflective tokens via GPT-4 API calls.

Pal, A. *et al.* (2022). Medmcqa: A large-scale multi-subject multi-choice dataset for medical domain question answering. In *Conference on Health, Inference, and Learning*.
Paszke, A. *et al.* (2019). Pytorch: An imperative style, high-performance deep learning library. *Advances in neural information processing systems*.
Rajbhandari, S. *et al.* (2020). Zero: Memory optimizations toward training trillion parameter models. In *SC20: International Conference for High Performance Computing, Networking, Storage and Analysis*. IEEE.

Prompt of Reflective token type Relevance (REL)

You'll be provided with an instruction, along with evidence and possibly some preceding sentences. When there are preceding sentences, your focus should be on the sentence that comes after them. Your job is to determine if the evidence is relevant to the initial instruction and the preceding context, and provides useful information to complete the task described in the instruction.
If the evidence meets this requirement, respond with **[Relevant]**; otherwise, generate **[Irrelevant]**.

Instruction: Based on the provided genetic information, classify whether the mutation described predisposes carriers to breast cancer. The provided dataset is a genetic report showing a mutation in the BRCA1 gene.
Evidence: TBRCA mutations: is everything said? BACKGROUND: Mutations in the BRCA1 and BRCA2 genes constitute a risk factor for breast cancer development. BRCA mutation research has been an active field since the discovery of the genes, and new mutations in both genes are constantly described and classified according to several systems. AIM: We intend to provide an overview of the current state of BRCA1 and BRCA2 mutation description and classification. We wanted to know whether there was a trend towards a more frequently described mutation type and what the proportion of pathogenic mutations was. RESULTS: We found that, although new mutations are described each year as reflected in current database records, very few of them are reported in papers. Classification systems are highly heterogeneous and a consensus among them
Rating: [Relevant]
Explanation: The evidence mentions BRCA1/2 mutation probability models and their potential role in predicting breast cancer risk, directly relevant to the instruction about classifying the predisposition to breast cancer based on a mutation.

Instruction: {Example Instruction}
Evidence: {Example Evidence}
Rating:

Table S6. Prompt of Relevant Reflective token. We use 1-shot to generate reflective tokens via GPT-4 API calls.

Singhal, K. *et al.* (2022). Large language models encode clinical knowledge. *arXiv preprint arXiv:2212.13138*.

Touvron, H. *et al.* (2023). Llama 2: Open foundation and fine-tuned chat models. *arXiv preprint arXiv:2307.09288*.

Wolf, T. *et al.* (2019). Huggingface's transformers: State-of-the-art natural language processing. *arXiv preprint arXiv:1910.03771*.

Prompt of Reflective token type Supportive (SUP)

You will receive an instruction, evidence, and output, and optional preceding sentences. If the preceding sentence is given, the output should be the sentence that follows those preceding sentences. Your task is to evaluate if the output is fully supported by the information provided in the evidence, and provide explanations on your judgement
Use the following entailment scale to generate a score:
[**Fully supported**] - All information in output is supported by the evidence, or extractions from the evidence. This is only applicable when the output and part of the evidence are almost identical.
[**Partially supported**] - The output is supported by the evidence to some extent, but there is major information in the output that is not discussed in the evidence. For example, if an instruction asks about two concepts and the evidence only discusses either of them, it should be considered a [Partially supported].
[**No support / Contradictory**] - The output completely ignores evidence, is unrelated to the evidence, or contradicts the evidence. This can also happen if the evidence is irrelevant to the instruction.
Make sure to not use any external information/knowledge to judge whether the output is true or not. Only check whether the output is supported by the evidence, and not whether the output follows the instructions or not.

Instruction: Ask about your symptoms and potential cardiovascular-related issues. I've been feeling intense discomfort in my chest during physical exertion. It sometimes shoots up to my left shoulder. There's also some mild shortness of breath.
Output: Based on the symptoms you have described, it is important to consider potential cardiovascular-related issues. Chest discomfort during physical exertion, shooting pain to the left shoulder, and mild shortness of breath can be indicative of a heart problem. These symptoms could potentially be related to angina, which is chest pain or discomfort caused by reduced blood flow to the heart. It commonly occurs during exercise or periods of stress. Another possibility is a myocardial infarction (heart attack), where there is a blockage in one of the coronary arteries supplying blood to the heart muscle. Given the seriousness of these symptoms, I would strongly advise you to seek immediate medical attention and consult with a healthcare professional for a proper evaluation and diagnosis. They will be able to conduct a thorough examination, ask additional questions about your medical history, possibly order tests such as an electrocardiogram (ECG) or stress test, and provide appropriate treatment recommendations based on their findings.
Evidence: Disparities in Awareness of Myocardial Infarction and Stroke Symptoms and Response Among United States– and Foreign-Born Adults in the National Health Interview Survey. Knowledge of MI symptoms was assessed based on responses to the question, "Which of the following would you say are the symptoms that someone may be having a heart attack?" Responses were pain or discomfort in the jaw, neck, or back; feeling weak, lightheaded, or faint; chest pain or discomfort; pain or discomfort in the arms or shoulder; and shortness of breath. Similarly, knowledge of stroke symptoms was assessed by an individual's response to the question, "Which of the following would you say are the symptoms that someone may be having a stroke?" Responses were sudden numbness or weakness of face, arm, or leg, especially on one side; sudden confusion or trouble speaking; sudden trouble seeing in one or both eyes; sudden trouble walking, dizziness, or loss of balance; and sudden severe headache with no known cause. Respondents who answered "yes" to the 5 queries on MI and stroke symptoms were classified as knowing the symptoms of MI and stroke. Those who answered "no," "don't know," or refused to answer were classified as not knowing the symptoms.
Score: [Fully supported]
Explanation: The output correctly identifies potential cardiovascular-related issues including angina and myocardial infarction (heart attack) based on the described symptoms. The evidence supports these concerns, mentioning commonly recognized symptoms of a heart attack including chest pain or discomfort, shooting pain in the shoulder, and shortness of breath. The output also correctly advises seeking immediate medical attention and consultation with a healthcare professional, which is an appropriate advice given the severity of these conditions.

Instruction: {Example Instruction}
Preceding sentences: {Example Preceding entences}
Output: {Example Output}
Evidence: {Example Evidence}
Score:

Table S7. Prompt of Supportive Reflective token. We use 1-shot to generate reflective tokens via GPT-4 API calls.

Cases of Instruction used at Training Generator Model

No Retrieval

Instruction: Write a simple explanation of the human cardiovascular system suitable for a non-medical audience. <noinput>

Output: [No Retrieval] It consists of the heart, blood vessels, and blood. [No Retrieval] Think of the heart as a powerful pump that never stops working. [No Retrieval] Its main job is to pump blood throughout the body. [No Retrieval] The blood vessels are like roads or highways that carry the blood to different parts of the body. [No Retrieval] There are three types of blood vessels: arteries, veins, and capillaries. [No Retrieval] Arteries carry oxygen-rich blood away from the heart to the body's organs and tissues. [No Retrieval] Veins bring back oxygen-poor blood from the organs and tissues to the heart. [No Retrieval] Capillaries are tiny blood vessels that connect the arteries and veins, allowing oxygen and nutrients to pass through to the cells and removes waste products from them. [No Retrieval] Blood is the fuel that keeps everything running smoothly. [No Retrieval] It carries oxygen and nutrients from the lungs and digestive system to all the cells in your body. [No Retrieval] It also carries waste products, such as carbon dioxide, away from the cells so they can be eliminated. [No Retrieval] So, the heart pumps the blood, the blood vessels deliver it, and the blood carries important substances to where they're needed, keeping the body healthy. [No Retrieval] In summary, the human cardiovascular system is like a transportation network that transports blood, nutrients, and oxygen to all parts of our body and removes waste products. [No Retrieval] The heart acts as a pump, blood vessels are the roads, and blood is the fuel that keeps everything running smoothly. [Utility:5]

Retrieval (Fully Supported)

Instruction: Classify whether this description corresponds to Type 1 or Type 2 diabetes mellitus. The patient showed onset of symptoms during childhood, including excessive thirst, frequent urination, rapid weight loss, and stomach pain. The tests confirmed low insulin levels and absence of insulin resistance

Output: [Retrieval] <paragraph> beta cells have been destroyed. About two-thirds of body cells require insulin to absorb glucose and in its absence, they will not be able to use the glucose (blood sugar) for energy. Type 1 diabetes usually comes on abruptly, although the damage to the beta cells may begin much earlier. Typical signs of Type 1 diabetes are a great thirst, hunger, a need to urinate often, and loss of weight. To treat the disease, the person must inject insulin, follow a diet plan, exercise daily, and test blood glucose several times a day. Type 1 diabetes usually occurs in children and adults who are under age 30. This type of diabetes used to be known as "insulin-dependent diabetes," "juvenile diabetes," "juvenile-onset diabetes" and "ketosis-prone diabetes." - Diabetes mellitus </paragraph> [Relevant] Based on the description provided, this corresponds to Type 1 diabetes mellitus. [Fully supported] [No Retrieval] Type 1 diabetes mellitus is typically characterized by an early onset of symptoms, often during childhood or adolescence. [No Retrieval] Common symptoms include excessive thirst (polydipsia), frequent urination (polyuria), rapid weight loss, and abdominal pain. [No Retrieval] In addition, individuals with Type 1 diabetes typically have low insulin levels and no insulin resistance. [No Retrieval] Therefore, based on the symptoms and test results described, the answer is Type 1 diabetes mellitus. [Utility:5]

Retrieval (No Support / Contradictory)

Instruction: A seven year old child with recurrent chest infections and exocrine pancreatic insufficiency is suspected of having cystic fibrosis. Sweat chloride levels have been observed to be between 40-60 mmol/l on two separate occasions. Which of the following test should be performed next to suppo the diagnosis of Cystic fibrosis? What of the following is the right choice?
(A) Repeat sweat chloride levels on a different day
(B) Demonstrate an abnormal 72 hour fecal fat collection
(C) Demonstrate an abnormal nasal potential difference
(D) Demonstrate an abnormal F508 mutation by DNA analysis

Output: [Retrieval] <paragraph> to expert centers where bioassays of CFTR function like nasal potential difference measurement or intestinal current measurement can be done. Still, in some patients, despite symptoms compatible with CF and some indication of CFTR dysfunction (e.g. only intermediate sweat chloride value), diagnostic criteria are not met (e.g. only 1 CFTR mutation identified). For these subjects, the term CFTR related disorder (CFTR-RD) is used. Patients with disseminated bronchiectasis, congenital bilateral absence of the vas deferens and acute or recurrent pancreatitis may fall in this category. CF has a very wide disease spectrum and increasingly the diagnosis is being made during adult life, mainly in subjects with milder phenotypes. In many countries, nationwide CF newborn screening (NBS) has been introduced. In screen positive babies, the diagnosis of CF must be </paragraph> [Relevant] The final answer is (C). [No support / Contradictory] [Utility:4]

Table S8. Cases of Instruction used at Training Generator Model. The Critic LM C predicts retrieval of any evidence depending on the instruction. Given biomedical evidence, the model C predicts whether the original output supports the evidence or not.
